# Golden era of radiosensitizers

**DOI:** 10.3389/fvets.2024.1450776

**Published:** 2024-12-06

**Authors:** Jana Cizkova, Ondrej Jan Dolezal, Vojtech Buchta, Jan Pospichal, Vit Blanar, Zuzana Sinkorova, Anna Carrillo

**Affiliations:** ^1^Department of Radiobiology, Military Faculty of Medicine, University of Defence, Hradec Kralove, Czechia; ^2^Department of Clinical Subspecialties, Faculty of Health Studies, University of Pardubice, Pardubice, Czechia; ^3^Department of Nursing, Faculty of Health Studies, University of Pardubice, Pardubice, Czechia

**Keywords:** gold nanoparticles, radiotherapy, radiosensitizers, *in vivo*, tumor sensitizing

## Abstract

The past 30 years have brought undeniable progress in medicine, biology, physics, and research. Knowledge of the nature of the human body, diseases, and disorders has been constantly improving, and the same is true regarding their treatment and diagnosis. One of the greatest advances in recent years has been the introduction of nanoparticles (NPs) into medicine. NPs refer to a material at a nanometer scale (0.1–100 nm) with features (specific physical, chemical, and biological properties) that are broadly and increasingly used in the medical field. Their applications in cancer treatment and radiotherapy seem particularly attractive. In this field, inorganic/metal NPs with high atomic number Z have been employed mainly due to their ability to enhance ionizing radiation’s photoelectric and Compton effects and thereby increase conventional radiation therapy’s efficacy. The improvement NPs enable relates to their enhanced permeation ability and longer retention effect in tumor cells, capacity to reduce toxicity of commercially available cancer drugs through advanced NPs drug delivery systems, radiation sensitizers of tumors, or enhancers of radiation doses to tumors. Advanced options according to size, core, and surface modification allow even such multimodal approaches in therapy as nanotheranostics or combined treatments. The current state of knowledge emphasizes the role of gold nanoparticles (AuNPs) in sensitizing tumors to radiation. We have reviewed AuNPs and their radiosensitizing power during radiation treatment. Our results are divided into groups based on AuNPs’ surface modification and/or core structure design. This study provides a complete summary of the *in vivo* sensitizing effect of AuNPs, surface-modified AuNPs, and AuNPs combined with different elements, providing evidence for further successful veterinarian and clinical implementation.

## Graphical abstract

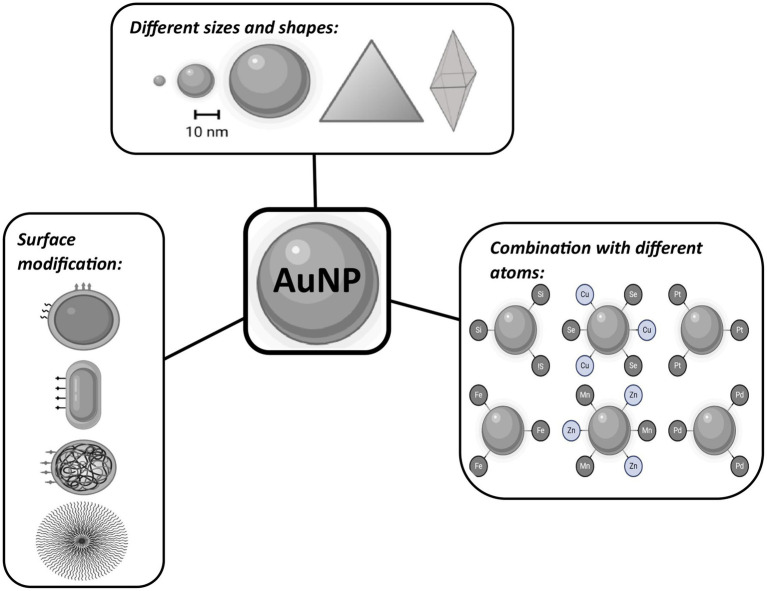

## 1 Introduction

Radiotherapy (RT) has been used for more than 100 years in oncological medicine for the local treatment of tumors (1). RT, as the name suggests, uses ionizing radiation (IR), mainly high-energy photon radiation (X-rays and gamma rays) and particle radiation (alpha, beta, electrons, protons, neutrons). Radiotherapy using an external beam or internal radioisotope emerged. New methods, such as intensity-modulated RT, image-guided RT, stereotactic RT, or particle therapy, have achieved more efficient tumor treatment and improved dose delivery to limit damage to normal tissues. Technical and computerized progress in beam modulation is only one of the numerous strategies for protecting normal tissue from unwanted radiation damage. Its therapeutic potential is often limited, however, by radioresistance of tumor cells (2–5). It is known that IR creates reactive forms of oxygen (ROS) due to water’s radiolysis and that this affects RT efficiency (6–9). The majority of solid tumors with low pO_2_ (hypoxic) are generally more radioresistant than are well-oxygenated tumors (normoxic) and necessitate treatment using larger doses (10–12). At higher dose levels, IR entails a greater risk of adverse effects on surrounding healthy tissue, although another additional strategy is needed to improve therapeutic results.

**Figure 1:**
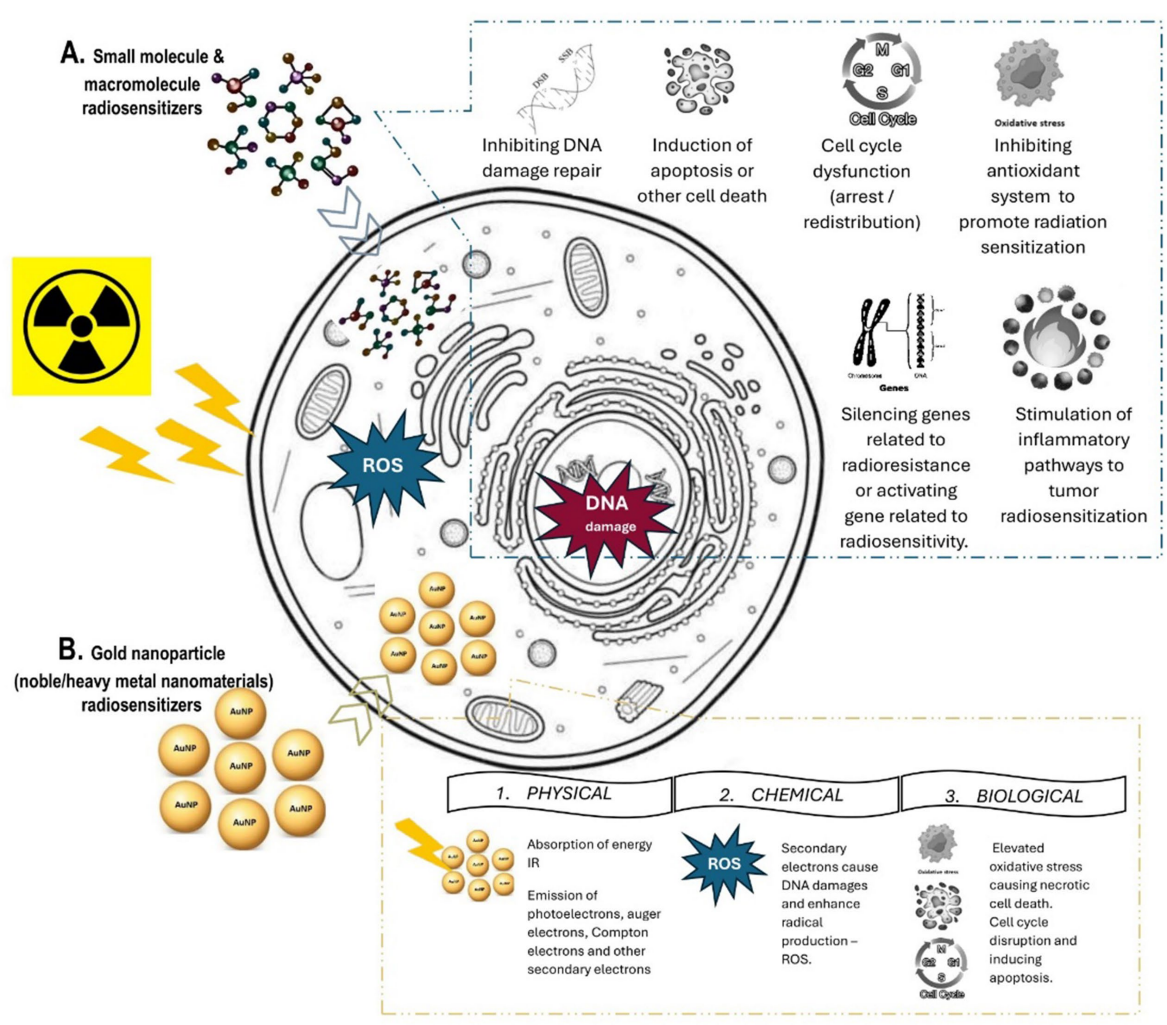
Radiosensitization: Mechanism of action of **(A)** small molecules and macromolecules radiosensitizers and **(B)** gold nanoparticle radiosensitizers.

One strategy is to use chemical or biological substances to reverse tumor resistance, increase tumor sensitization to IR, enhance normal tissues’ tolerance, and limit radiation dose deposition to the tumor volume. A wide range of obstacles add to the challenges of using RT to treat tumors, such as cancer stem cells, tumor heterogeneity, angiogenesis and vasculogenesis, metabolic changes, and various complications (10, 13, 14). One way to overcome these obstacles is to intensify the efficiency of RT by introducing a radiosensitizer (RS)—a substance or molecule that can increase the cytotoxic effects of radiation on cancer cells and improve radiotherapy results. The primary mechanism of RS action (Figure 1) involves one of the following possibilities (15–17):

- Inhibiting radiation-induced DNA damage repair, generating ROS that cause further oxidative damage, or modifying the tumor microenvironment to make it more conducive to radiation.
- Disrupting cell cycle and organelle function to enhance cytotoxicity, pushing cells toward apoptosis or mitotic failure after radiation exposure. This approach is beneficial for treating tumors resistant to radiation or poorly oxygenated.
- Silencing the expression of radioresistance genes or activating the expression of radiosensitive genes.

The first classification of RSs was made by Adams (18). He classified these into five categories according to their actions: (I) suppression of intracellular -SH groups or other endogenous radioprotective substances; (II) formation of toxic radiation products from radiolysis of the sensitizer; (III) inhibition of cellular repair processes after irradiation; (IV) structural incorporation of thymine analogs into intracellular DNA; and (V) oxygen mimics with electrophilic activity. This initial categorization of RSs based on the mechanisms of DNA damage and repair set the direction for future research and development of RS (18). Currently, RSs can be divided into three categories based on their structures: (I) small molecules—oxygen and its mimics, nitro compounds, and hypoxia-specific cytotoxins can enhance RT through free radical damage; (II) macromolecules, such as miRNAs, proteins, peptides, and oligonucleotides, can directly interact with vital proteins, conjugate with nanomaterials, bind and degrade mRNAs or DNAs; and (III) nanostructures—especially high atomic number materials that can absorb, scatter, and emit energy (16, 17). Radiosensitizing nanostructures comprise today one of the most significant candidates for boosting RT’s efficacy.

The expeditious development and application of nanoscale particles and other nanostructures (e.g., nanomachines, nanofibers for tissue engineering, nanoscale devices, and biosensors) has given rise to a new medical discipline, namely, nanomedicine. These groundbreaking innovations change the fundaments of disease diagnosis, monitoring, treatment, and prevention. In this field, liposomes, niosomes (non-ionic surfactant-based vesicles), dendrimers, mesoporous silica, and magnetic and metallic nanoparticles (NPs) are intensively produced (19). The discovery of nanotechnology led to the possibility of creating new RSs based on biomolecules or chemical elements with high atomic number Z. These elements contain large numbers of electrons in their atoms, which can absorb, scatter, and emit radiation energy (20, 21). The advantage of NPs is their rapid distribution in the body, low toxicity (9, 22, 23), and passive accumulation in tumor sites, termed the enhanced permeability and retention (EPR) effect (24–26). Nanoscience makes it possible today to prepare NPs of different sizes that pass the blood–brain barrier and gastrointestinal barrier (≈100 nm) (27), shapes that will fundamentally affect the interaction with the phospholipid bilayer (28), and surface functionalization using targeted molecules (29, 30).

Progressive research and the era of NPs have significantly affected today’s modern treatment opportunities in cancer therapy. Reportedly, just two of the numerous NP structures have reached the level of clinical trials for RT. Currently, 16 nano-based preparations for cancer therapy are approved by the US Food and Drug Administration (FDA), whereas more than 75 nanoformulations are now in clinical trials (31). One of the shining examples of NP radiosensitizer already used in patients with prostate adenocarcinoma is crystalline hafnium oxide NPs (NBTXR3, *Z* = 72; the size of NPs = 50 nm) whose clinicalTrials.gov identification number is NCT02805894. Among NBTXR3’s advantages are that it has no degradation and signaling activity in the organism, is inactive without radiotherapy, and is biologically safe. After IR exposure, intratumor (i.t.) application of NBTXR3 can amplify tumor radiation energy during RT—that is, it acts as a radiological enhancer. NBTXR3 is widely used as a radiological enhancer in various malignancies (32–34). Other NP structures are activation and guiding of irradiation by X-ray or AGuIX^®^ gadolinium-based NPs (*Z* = 64, size of NPs = 5 nm) in patients with brain metastases for management of stereotactic radiation for more effective treatment whose clinicalTrials.gov identification number is NCT04899908. AGuIX® NPs not only can amplify the radiation dose but also are capable of magnetization, which is why they are also used for magnetic resonance imaging (MRI) (35, 36). On the same principle, another clinical study (clinicalTrials.gov identification number is NCT04682847) used MRI–superparamagnetic iron oxide nanoparticles (SPIONs) radiotherapy for primary and metastatic hepatic carcinoma. SPIONs have unique superparamagnetic properties in the presence of a magnetic field suitable for imaging but also for photothermal therapy (PTT) or magnetic hyperthermia (induced by alternating magnetic field) (37).

Among other materials, gold nanoparticles (AuNPs) have been studied intensively. Advanced features of AuNPs include a high Z number (*Z* = 79) available to deposit a high volume of IR energy, excellent biocompatibility, low toxicity, great surface area volume suitable for surface modification, and improved and enhanced permeability and retention effect in tumor sites with low systemic clearance. Also, these NPs can serve as contrasting agents for imaging modalities due to their high atomic number. However, it is important to note that the size, shape, and surface chemistry of these NPs have different effects on their biodistribution and biological properties (38–40). Currently, three preparations of AuNPs are under clinical testing for cancer therapy: CYT-6091 (Aurimune), NU-0129, and AuroShell. CYT-6091 (Aurimune) is a colloidal Au core delivery system with rhTNF for tumor targeting (clinicalTrials.gov identification numbers are NCT00356980 and NCT00436410, respectively). NU-0129 is an Au core modified with RNA-based spherical nucleic acid to inhibit glioblastoma (clinicalTrials.gov identification number is NCT03020017). AuroShell is a silica core coated with a gold shell for PTT (clinicalTrials.gov ID: NCT03020017) (39). Nonetheless, none of these preparations are primarily used due to the radiosensitizing properties of AuNPs. It has been extensively explored that AuNP radiosensitization (Figure 1), as true of every noble/heavy metal NP, is due to the absorption of energy from RT. Consequently, this energy can further locally ionize surrounding tissue, mainly by emitting scattered photons, photoelectrons, Compton electrons, Auger electrons, or fluorescence photons. This specific physical phase of radiosensitization is followed by the secondary chemical (water radiolysis, leading to the production of additional ROS) and the biological (further damage to cellular structures causing oxidative stress, mitochondrial dysfunction, cell-cycle effect, and DNA repair inhibition) phases in the targeted tissue (17, 41, 42). We aim to summarize the possibilities of AuNPs in cancer treatment as a radiosensitizer. The uniqueness of this study is that it describes and compares the biological properties not just of AuNPs but also of surface-modified AuNPs and combinations of AuNPs with different elements because all these modifications alter the radiosensitizing power. This review focuses on the various aspects of AuNPs and highlights the application opportunities making this the next suitable NP for clinical trials.

## 2 Materials and methods

We conducted a literature review conforming to the Preferred Reporting Items for Systematic Reviews and Meta-Analyses (PRISMA) standards. The PRISMA framework contains key guidelines to improve the clarity and quality of systematic reviews and meta-analyses. The PRISMA 2020 statement consists of a 27-point checklist with detailed recommendations and revised flowcharts from the previous form. The checklist items were followed to define the methodology and eligibility criteria, describe the sources of information, and outline the full search strategy and the study selection process. The final steps are the processes of data collection and its presentation. We did not attempt to summarize the results of the different studies quantitatively or statistically (i.e., to create a meta-analysis).

### 2.1 Inclusion and exclusion criteria

To map relevant publications, exclusion and inclusion criteria were established. To be included, studies had to consider the application of IR on the tumor side to verify the radiosensitizing effect of AuNPs *in vivo*. Studies dealing with the synthesis and characterization of AuNPs (EC1) or studying effects other than radiosensitizing of AuNPs or *in silico*/*in vitro* experiments (EC2) were excluded. Studies were excluded if they evaluated the effects of AuNPs on diseases other than malignancies (EC3). Also, studies were omitted when gold elements were added to the NPs’ surface, or AuNPs were used as contrasting imaging agents (EC3). Also, IR had to be used in the research on the radiosensitizing effect of AuNPs. No other radiation sources were acceptable for review (EC4).

### 2.2 Search methodology

The PubMed, Medline, Scopus, and Google Scholar databases were used for publication search, but to accumulate sufficient material, published articles also were searched manually in reference lists. This investigation was last performed in January 2023. The database searches had no time limitations. We used various search term combinations that include the following:

- “gold nanoparticles,” “gold nanoparticle,” and “golden nanoparticles.”
- “sensitizing,” “sensitizers,” “radiosensitizer,” and “radiosensitizing.”
- “radiotherapy” and “radiation therapy.”

**Figure 2:**
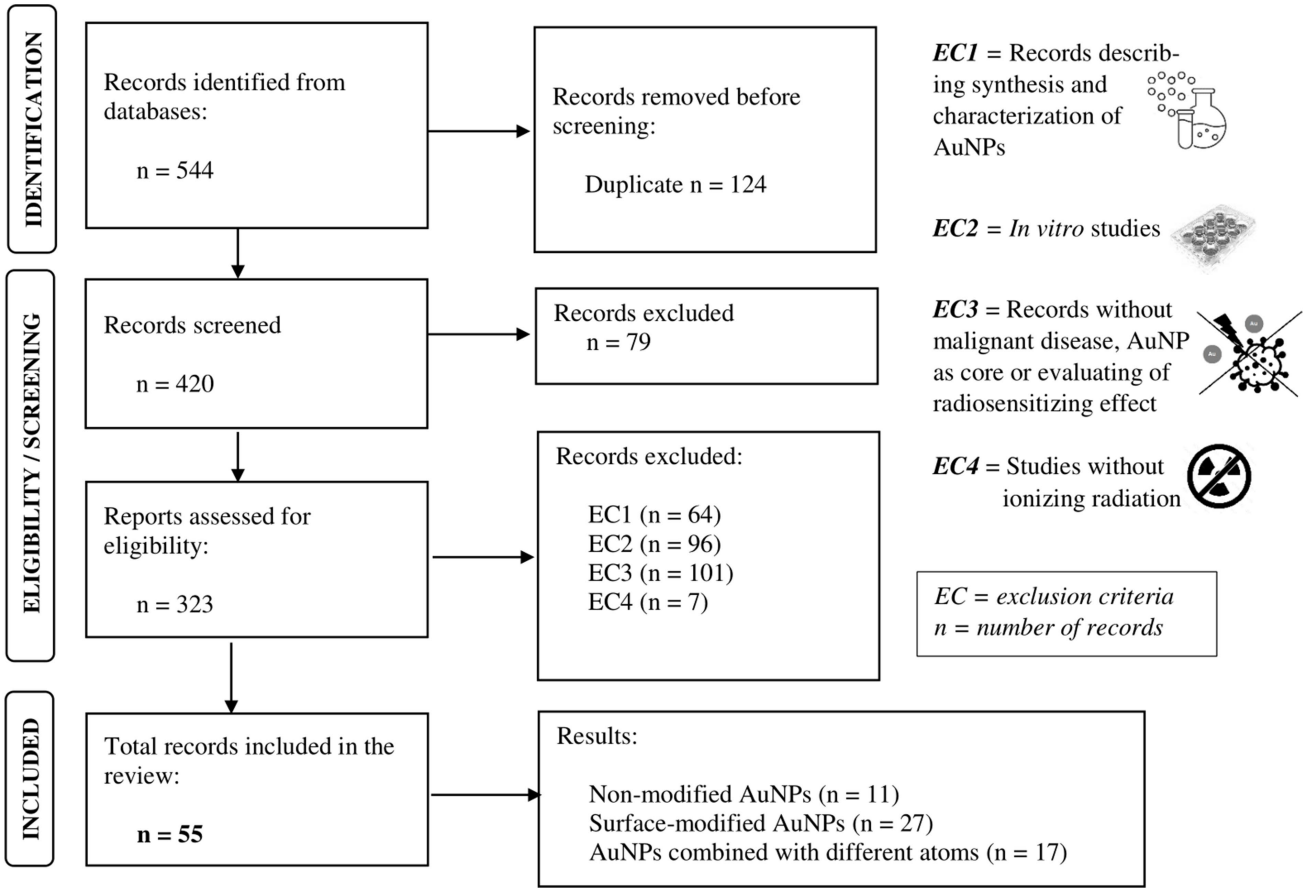
Preferred Reporting Items for Systematic Reviews and Meta-Analyses (PRISMA) flow diagram for publications search.

Through the literature search (Figure 2), using the combination of Boolean operators “AND” and “OR,” searching in “[Title/Abstract]” of each article, a total of 544 relevant articles were identified.

### 2.3 Search summary

Although we aimed to summarize the potential of AuNPs for further clinical application, only *in vivo* studies were included (Figure 2). This selection of studies was performed by two independent evaluators. In case of disagreement, a third evaluator was included. The total number of included studies is *n* = 56. Subsequently, based on their chemical properties, AuNPs were divided into three groups: non-modified AuNPs (*n* = 11 studies), surface-modified AuNPs with a chemical molecule or compound to improve their intrinsic physical, chemical, and/or biological characteristics (*n* = 27 studies), and AuNPs combined with different chemical elements (*n* = 18 studies).

### 2.4 Data collection

An overview of the individual studies was then provided in each section of the studies. To describe the radioprotective effect of AuNPs, the following data were compiled from the reports: form, size, and concentration of AuNPs tested; radiation doses used, *in vivo* laboratory model and analytical methods listed; and main conclusion of each study considering further toxicity. This information is described in the following section and summarized in Tables 1–3 for ease of reference.

**Table 1:** Summary of studies involving non-modified AuNPs.

First author (year)	Shape	Size and concentration	IR dose	*In vivo* analysis	Result	References
Hainfeld (2004)	Spherical	1.9 ± 0.1 nm (2.7 or 1.35 g/kg)	30 and 26 Gy	Gold analysis of tissues, imaging, survival analysis, radiation therapy—radiosensitization, and blood tests	Longer survival, no toxicity damage	Hainfeld et al. (41)
Chang (2008)	Spherical	13 nm (1 g/kg)	25 Gy	Biodistribution, survival analysis, and radiation therapy—radiosensitization	Longer survival, increased apoptosis of TC, no toxicity damage	Chang et al. (45)
Chen (2015)	Spherical (BSA)	28 nm (1.3 g/kg)	2 × 5 Gy	Biodistribution and toxicology studies and radiation therapy—radiosensitization	Tumor regression, no toxicity damage	Chen et al. (46)
Hassan (2020)	Spherical (citrate)	50 nm (90 mg/kg)	5 Gy	Immunohistochemistry, TUNEL assay, and radiation therapy—radiosensitization	Tumor growth inhibition, increased apoptosis of TC, no toxicity damage	Hassan et al. (47)
Liu (2018)	Spherical (BSA)	187, 50, and 8 nm (4 mg/kg)	5 Gy	Biodistribution, survival analysis, immunohistochemistry, and radiation therapy—radiosensitization	Longer survival, tumor growth inhibition—depending on NP’s size, increased apoptosis of TC, no toxicity damage	Liu et al. (48)
Janic (2021)	Spherical	4 and 14 nm (2 mg/kg)	15 Gy	Toxicity, radiation therapy—radiosensitization, immunological analysis, and survival analysis	Longer survival, tumor growth inhibition—depending on NP’s size, minimal toxicity (weight)	Janic et al. (49)
Bhattarai (2017)	Nanotriangles	61.51 ± 2.91 nm (2.7 mg/kg)	4 and 6 Gy	Toxicity, biodistribution, radiation therapy—radiosensitization, and survival analysis	Longer survival, tumor growth inhibition, infiltrates in liver and spleen	Bhattarai et al. (50)
Mulgaonkar (2017)	Hollow core	120 nm (2.8 mg/kg)	10 Gy	Radiation therapy—radiosensitization, survival analysis, and MRI and CT imaging	Longer survival, tumor growth inhibition, no toxicity damage	Mulgaonkar et al. (51)
Chuang (2019)	Nanodandelions	73 ± 10 nm (1 mg per mouse)	16 Gy	Tumor growth and radiation therapy—radiosensitization	Tumor growth inhibition, no toxicity damage	Chuang et al. (52)
Zhang (2020)	Spherical	40 nm (1 mg/kg)	4 Gy	Multimodal tumor imaging and combination PTT + radiation therapy—radiosensitization	Tumor growth inhibition, combined treatment efficacy, no toxicity damage	Zhang et al. (54)
Li (2020)	Nanobipyramids (PEG)	38 ± 2 nm (10 or 15 mg/kg)	5 and 8 Gy	Immunofluorescence staining for hypoxia and combination PTT + radiation therapy—radiosensitization	Tumor growth inhibition, combined treatment efficacy, no toxicity damage	Li et al. (55)

BSA, bovine serum albumin; CT, computed tomography; IR, ionizing radiation; MRI, magnetic resonance imaging; PEG, polyethylene glycol; PTT, photothermal therapy; RT, radiotherapy; TUNEL, terminal deoxynucleotidyl transferase dUTP nick-end labeling; TC, tumor cells.

**Table 2:** Summary of studies involving surface-modified AuNPs.

First author (year)	Shape	Size and concentration	Surface modification	IR dose	*In vivo* analysis	Result	References
Chattopadhyay (2013)	Spherical	30 nm (4.8 mg/g)	Trastuzumab	11 Gy	Tissue toxicity, targeted tumor delivery, image-guided radiation therapy—radiosensitization	Tumor growth inhibition, no toxicity damage	Chattopadhyay et al. (56)
Zhang (2019)	Spherical	78.3 nm, 75.02 nm (10 μg/tumor)	DMMA + cisplatin	4 Gy	Tumor uptake study, tumor microenvironment, synergistic chemoradiotherapy, H&E staining	Tumor growth inhibition, combined treatment efficacy, no toxicity damage	Zhang et al. (57)
Chen (2020)	Nanorods	122 nm (1 mg/mL)	Doxorubicin	5 and 10 Gy	Synergistic chemoradiotherapy—radiosensitization	Tumor growth inhibition, combined treatment efficacy, increased apoptosis of TC, little major organ toxicity	Chen et al. (58)
Shi (2016)	Spherical	2.77 ± 0.69 nm (i.t.: 73.36 μg/μl i.v.: 36.63 μg/μl)	Tiopronin	10 Gy	Radiation therapy—radiosensitization, tumor uptake, survival analysis	Longer survival, tumor growth inhibition, no toxicity damage	Shi et al. (59)
Jia (2019)	Nanoclusters	2 nm (50 μM)	Levonorgestrel	4 Gy	Biodistribution, H&E staining, radiation therapy—radiosensitization	Tumor growth inhibition, no toxicity damage	Jia et al. (60)
Wang (2021)	Spherical	58 nm (200 μg/mouse)	8-Hydroxy quinoline	2 × 4 Gy	Imaging, biodistribution, radiation therapy—radiosensitization, immunofluorescence staining- hypoxia	Tumor growth inhibition, no toxicity damage	Wang et al. (61)
Li (2021)	Spherical	12–15 nm (4 mg/kg)	Glucose	10 Gy	Radiation therapy—radiosensitization, RT-qPCR, immunofluorescence staining- hypoxia	Tumor growth inhibition, no toxicity damage	Li et al. (62)
Jia (2021)	Nanoclusters	2.59 nm (n.m.)	D-fructose	4 Gy	Biodistribution, H&E staining, radiation therapy—radiosensitization	Tumor growth inhibition, no toxicity damage	Jia et al. (63)
Xu (2019)	Nanocages	193.3 ± 8.1 nm (10 mg/kg)	HA	6 Gy	Biodistribution and targeting efficiency, imaging, combination PTT + radiation therapy—radiosensitization	Tumor growth inhibition, combined treatment efficacy, no toxicity damage	Xu et al. (64)
Hua (2021)	Nanoclusters	53.1 ± 11.7 nm (1 mg/kg)	CS + ICG	6 Gy	Biodistribution, PTT + radiation therapy—radiosensitization, H&E and TUNEL staining	Tumor growth inhibition, combined treatment efficacy, metastasis inhibition, minimal toxicity damage	Hua et al. (65)
Zhang (2014)	Nanoclusters	3.00 ± 1.02 nm (10 mM)	GTH	5 Gy	Biodistribution, tumor uptake, radiation therapy—radiosensitization, histopathology and blood biochemistry examinations	Tumor growth inhibition, improved EPR effect, no toxicity damage	Zhang et al. (66)
Zhang (2015)	Nanoclusters	2.8 nm (5.9 mg/kg)	GTH	5 Gy	Biodistribution, tumor uptake, radiation therapy—radiosensitization, histopathology and blood biochemistry examinations	Tumor growth inhibition, no toxicity damage	Zhang et al. (67)
Zhang (2018)	Nanoclusters	4.0–6.3 nm (n.m.)	Histidine	6 Gy	Cell cycle analysis, radiation therapy—radiosensitization	Tumor growth inhibition, no toxicity damage	Zhang et al. (70)
Liang (2017)	Nanoclusters	3.2 ± 0.54 nm (20 mM)	cRAD and cRGD peptides	6 Gy	CT imaging, biodistribution, radiation therapy—radiosensitization	Tumor growth inhibition, no toxicity damage	Liang et al. (68)
Dong (2021)	Spherical	10.6 nm (2 mg/kg)	iRGD peptide +*α*-difluoro methylornithine	2 × 4 Gy, 2 × 8 Gy	Fluorescent imaging, biodistribution, survival analysis, blood–brain barrier penetration, cell cycle regulation, radiation therapy—radiosensitization	Longer survival, tumor growth inhibition, no toxicity damage	Dong et al. (69)
Luo (2019)	Nanoclusters	5.0 nm (30 μg /mouse)	PSMA-1 peptide	6 Gy	CT imaging, tumor targeting, biodistribution, inductively coupled plasma mass spectrometry, radiation therapy—radiosensitization	Tumor growth inhibition, no toxicity damage	Luo et al. (71)
Nicol (2018)	Spherical	36.6 ± 3.07 nm (6 mg/kg)	RME and H5WYG peptides	4 Gy	Radiation therapy—radiosensitization, survival analysis	No effect on survival, tumor growth inhibition, minimal toxicity (weight)	Nicol et al. (72)
Ma (2017)	Nanospikes	54 ± 9 nm (400 μg/μl)	TAT cell-penetrating peptide	6 Gy	Radiation therapy—radiosensitization, fluorescence staining—autophagy	Tumor growth inhibition, minimal toxicity (weight)	Ma et al. (73)
Liu (2017)	Nanoclusters	16.3 nm (n.m.)	Anti-RhoJ antibody	5 Gy	Biodistribution, radiation therapy—radiosensitization, immunofluorescence, imaging, pathological study—angiogenesis	Tumor growth inhibition, angiogenesis inhibition, low toxicity damage	Liu et al. (74)
Gal (2022)	Spherical	20 nm (6 mg/kg)	Insulin, cetuximab	10 Gy	Micro-CT imaging, immunohistochemistry—angiogenesis, survival analysis, inductively coupled plasma-optical emission spectrometry analysis	Longer survival, tumor growth inhibition, combined treatment efficacy, angiogenesis inhibition, no toxicity damage	Gal et al. (75)
Wolfe (2015)	Nanorods	31 nm (400 μg)	Goserelin	5 Gy	Biodistribution, radiation therapy—radiosensitization	Tumor growth inhibition, no toxicity damage	Wolfe et al. (76)
Ghahremani (2018)	Nanoclusters	15.2 nm (8 mg/kg)	AS1411 aptamer	6 Gy	Biodistribution, radiation therapy—radiosensitization, survival analysis, histopathology examination and blood biochemistry	Longer survival, tumor growth inhibition, no toxicity damage	Ghahremani et al. (77)
Kefayat (2019)	Nanoclusters (BSA)	5.5 ± 0.4 nm (10 mg/kg)	FA	6 Gy	Biodistribution, radiation therapy—adiosensitization, survival analysis, histopathology and blood biochemistry examinations, immunostaining	Longer survival, tumor growth inhibition, increased apoptosis of TC, no toxicity damage	Kefayat et al. (78)
Cheng (2019)	Nanoprobes	64.0 nm (2 mg/kg)	FA + photolabile diazirine group	4 Gy	CT imaging, survival analysis, flow cytometry, combination laser (405 nm) + radiation therapy—radiosensitization	Longer survival, Tumor growth inhibition, increased apoptosis of TC, combined treatment efficacy, no toxicity damage	Cheng et al. (79)
Ding (2020)	Spherical	n.m. (5 mg/kg)	FA + 1,3-cyclo hexanedione	8 Gy	Radiation therapy—radiosensitization, CT imaging, biodistribution, H&E and immunofluorescent staining	Tumor growth inhibition, increased apoptosis of TC, no toxicity damage	Ding et al. (80)
Masood (2012)	Nanorods	50–70 nm (1 mg/kg)	SphK1 gene	1 Gy 2× weekly	Radiation therapy—radiosensitization, H&E and immunohistochemical staining	Tumor growth inhibition, increased apoptosis of TC, no toxicity damage	Masood et al. (81)
Yang (2021)	Dendrimers	175.7–206.7 nm (5 mg/kg)	HIF-1α gene	6 Gy	Radiation therapy—radiosensitization, H&E, immunohistochemical and immunofluorescence staining, biodistribution, anti-metastasis study	Tumor growth inhibition, metastasis inhibition, no toxicity damage	Yang et al. (82)

BSA, bovine serum albumin; cRAD, arginine-alanine-aspartic acid peptide; cRGD, arginine-glycine-aspartic acid peptide; CS + ICG, chitosan and fluorescent green dye; CT, computed tomography; FA, folic acid; GTH, glutathione; HA, hyaluronic acid; H&E, hematoxylin–eosin; n.m., not mentioned; TC, tumor cells.

**Table 3:** Summary of studies combining AuNPs with different atoms.

First author (year)	Shape	Size and concentration	Other atom (s)	Surface modification	IR dose	*In vivo* analysis	Result	References
Zhao (2016)	Nanorods	80.94 ± 2.69 *(diameter changed over time)* (1 mg/g)	Si	cRGD-peptide	10 Gy	Biodistribution, radiation therapy—radiosensitization	Tumor growth inhibition, no toxicity damage	Zhao et al. (83)
Chiang (2021)	Core–shell	20 ± 6 nm (100 μg/μl)	Si	HA	2 and 10 Gy	Safety, efficacy, survival analysis, H&E staining, radiation therapy—radiosensitization	Longer survival, tumor elimination, no toxicity damage	Chiang et al. (84)
Wang (2023)	Spherical	30 nm (n.m.)	Si	Toripalimab, bevacizumab	5 Gy	Radiation therapy—radiosensitization	Tumor growth inhibition, no toxicity damage	Wang et al. (85)
McQuade (2015)	Micells	100 nm (400 mg/kg)	Fe	–	6 Gy	Blood distribution, clearance, and tumor delivery, toxicity studies, imaging, radiation therapy—radiosensitization	Tumor growth inhibition, no toxicity damage	McQuade et al. (87)
Chen (2017)	Core–shell (PEG)	100 nm (2 mg/kg)	Fe	–	4 Gy	Tumor magnetic resonance/ photoacoustic imaging, thermo-radiation therapy—radiosensitization, blood analysis	Tumor growth inhibition, combined treatment efficacy, no toxicity damage	Chen et al. (88)
Nosrati (2020)	Heterodimer	138.4 nm (6 mg/mL)	Fe	FA	2, 4, and 6 Gy	Safety analysis and LD_50_, radiation therapy—radiosensitization, histopathology analysis	Tumor growth inhibition, no toxicity damage	Nosrati et al. (89)
Hua (2021)	Nanoclusters	100 nm (10 mg/kg)	Fe	cRGD-peptide	4 Gy	Biodistribution, imaging, radiation therapy—radiosensitization, toxicity	Tumor growth inhibition, no toxicity damage	Hua et al. (90)
Chang (2017)	Nanorods	120 nm (2 mg/kg)	Se	–	4 Gy	Photoacoustic imaging, MRI, acute toxicity, biodistribution, radiation therapy—radiosensitization	Tumor growth inhibition, no toxicity damage	Chang et al. (91)
Huang (2019)	Nanocrystals	17.6 ± 1.4 nm (2.5 mg/kg)	Se, Cu	–	6 Gy	Photoacoustic imaging, CT imaging, single-photon emission CT imaging, PTT+ radiation therapy—radiosensitization, histology analysis	Tumor growth inhibition, combined treatment efficacy, no toxicity damage	Huang et al. (92)
Yi (2016)	Core–shell (PEG) (3.8 mg/kg)	50 nm	Mn	–	6 Gy	MRI, hypoxia tumor analysis, radiation therapy—radiosensitization, blood analysis and histology examinations, blood circulation and biodistribution study	Tumor growth inhibition, no toxicity damage	Yi et al. (93)
Chen (2017)	Nanoclusters (BSA) (37.9 mg/kg)	60 nm	Mn	–	6 Gy	Immunofluorescence staining, imaging, radiation therapy—radiosensitization, biodistribution study	Tumor growth inhibition, no toxicity damage	Chen et al. (94)
Lin (2021)	Core-shell (1 mg/mL)-	100 nm	Mn	GTH	4 and 6 Gy	Imaging, radiation therapy—radiosensitization	Tumor growth inhibition, no toxicity damage	Lin et al. (95)
Li (2016)	Core–shell-shell (150 mg/kg)	30–40 nm	Mn, Zn	–	4 Gy	Blood circulation and biodistribution study, MRI, radiation therapy—radiosensitization, histological examination, *γ*-H2AX staining	Tumor growth inhibition, no toxicity damage	Li et al. (96)
Yang (2021)	Spherical (PEG) (20 mg/kg)	150 ± 20 nm	Pt	–	8 Gy	Pharmacokinetic parameters and biodistribution, radiation therapy—radiosensitization	Tumor growth inhibition, no toxicity damage	Yang et al. (97)
Cheng (2018)	Nanoprobes (PEG) (12 mg/kg)	79.6 ± 5.4 nm	Ti	–	10 Gy	Radiation therapy—radiosensitization, biodistribution, histological examination	Tumor growth inhibition, no toxicity damage	Cheng et al. (98)
Liu (2021)	Core−shell (PEG) (4 mg/kg)	10.4 ± 1.6 nm,	Pd	–	4 Gy	Pharmacokinetics and biodistribution, radiation therapy—radiosensitization	Tumor growth inhibition, no toxicity damage	Liu et al. (99)
Xiang (2020)	Core–shell (PVP) (50 μg)	30 ± 10 nm	Pd	–	2 × 5 Gy	Photothermal imaging and heating, synergetic PTT + radiation therapy—radiosensitization	Tumor growth inhibition, combined treatment efficacy, no toxicity damage	Xiang et al. (100)

BSA, bovine serum albumin; cRGD, arginine-glycine-aspartic acid; CT, computed tomography; FA, folic acid; GTH, glutathione; HA, hyaluronic acid; MRI, magnetic resonance imaging; n.m., not mentioned; PEG, polyethylene glycol; PTT, photothermal therapy; PVP, polyvinyl pyrrolidone; RT, radiotherapy.

## 3 Results

### 3.1 Non-modified AuNPs

As mentioned, AuNPs with high Z numbers are suitable as radiosensitizers and increase the dose given to a tumor during radiation therapy. The majority of the studies were done with spherical shapes of AuNPs; exceptions are mentioned in Table 1. This group also included AuNPs that were only surface modified with polyethylene glycol (PEG), bovine serum albumin (BSA), or citrate to improve their biological half-life in the body, for better retention and solubility, and/or to avoid aggregation but did not change properties of the NPs (43, 44).

The first pioneering study that dealt with the use of AuNPs (*d* = 1.9 ± 0.1 nm) to enhance the effects of RT is that of Hainfeld et al. (41) in Balb/c mouse-bearing mammary gland tumor (EMT-6). AuNPs radiosensitization was manifested 1 month after irradiation when, in the RT group (30 Gy), tumors grew to as much as 5× their original size. At the same time, mice that received AuNPs had no visible tumors, and one even had a shrinking tumor. Responses lasted more than 1 year after IR (26 Gy). The most significant accumulation of AuNPs was in the tumor (7.0 ± 1.6 min) and muscles (5.3 ± 0.6 min). AuNPs are rapidly excreted from the muscles (t_1/2_ = 24.2 ± 2.6 min). Five minutes after application, the ratio of gold in the tumor to that in the muscle was 3.5:1 (41). Further study by Chang et al. (45) also addressed the enhancement of the effect of RT using AuNPs in C57BL/6 with melanoma. The experiment revealed a slowing in tumor growth in the RT (25 Gy) and AuNPs + RT groups. AuNPs had no antitumor/radiosensitizing effect. The highest numbers of apoptotic cancer cells were in the AuNPs + RT group. This study confirmed similar biokinetics of AuNPs and an accumulation ratio of 6.4:1 between the tumor and the tumor surrounding the muscles. AuNPs also had a denser concentration in the spleen and liver, accumulating through the reticuloendothelial system (45). In the study by Chen et al. (46), they dealt with radiosensitizing effects of AuNPs surface-covered with BSA in nude mice bearing U87 glioblastoma. BSA-AuNPs (28 nm) alone did not affect tumor growth. The BSA-AuNPs+RT (sequentially by 3 Gy and 2 Gy at 2 and 24 h treatment) group demonstrated the most significant tumor regression. Cell swelling, nuclear pyknosis, and cell debris were found in the BSA-AuNPs + RT groups. Combining RT with BSA-AuNPs resulted in the most significant reduction in body weight gain of mice across all groups. BSA-AuNPs alone did not affect weight gain in mice, and IR radiation alone suppressed weight gain. Clearance of AuNPs started within 2 h after application. Toxicity analysis showed no critical effects on vital organs during 24 h, but later they began to show a long-term impact (46). In a study by Hassan et al. (47), they compared the radiosensitizing effects of AuNPs with titanium TiNPs in immunodeficient mice bearing pancreatic carcinoma (MIA PaCa-2) cells. The group of TiNPs + RT (*d* = 50 nm, *D* = 5 Gy) mice showed better radiosensitization effects to prevent and slow the growth of tumor tissues compared to RT and AuNPs + RT (*d* = 50 nm) groups. None of the mice died during the 55-day observation period, and none of them showed apparent weight loss. Without radiation, AuNPs and TiNPs slightly increased the number of apoptotic cells. Pure AuNPs without radiation did not increase apoptosis (47).

Other studies compare the radiation effect of AuNPs of different sizes. Liu et al. (48) studied the radiosensitizing effects of different AuNPs (187, 50, and 8 nm) on the murine hepatocellular carcinoma (H22) model. The results showed that tumors in all sizes of AuNPs + RT grew more slowly compared to those in the other groups. Tumors in the small AuNPs + RT and medium AuNPs + RT groups had slower tumor tissue growth than those seen in the other groups. Small and medium AuNPs have a radiosensitizing effect in RT and more prolonged survival, while 187 nm AuNPs have only a limited effect. All groups had no abnormal physiological responses. The mice showed no toxicity during this experiment in the thymus, spleen, liver, or kidney (48). In a study by Janic et al. (49), they examined therapeutic enhancement of the effect of RT and immunomodulation of AuNPs in a xenograft of human breast cancer (triple negative). Mice were administered AuNPs of size 4 or 14 nm and combined with RT of *D* = 15 Gy. Mice with RT and RT + 4 nm or 14 nm AuNPs had delayed tumor growth and a significant difference–a radiosensitizing effect (*p* < 0.05)—was observed in both groups with AuNPs on day 30. Mice had changes in body weights, but the magnitude was relatively small at less than ±10% over the study. Survival analysis showed that mice with 4 nm AuNPs + RT and 14 nm AuNPs + RT had even higher survival (*p* < 0.05). A significant enhancement of the effect of RT for survival was achieved by the combination of 14 nm AuNPs + RT but was not observed in the group of 4 nm AuNPs. Intratumor distribution on day 26 revealed 14 nm AuNPs dispersed more evenly in the tissue, while 4 nm AuNPs gathered in visible clusters (49).

The structure and shape of AuNPs are also significant features that can affect their biological and sensitizing abilities. In the study by Bhattaral et al. (50), they looked at the radiosensitizing effects of gold nanotriangles (AuNTs) in mice bearing U87MG glioblastoma. The AuNTs (61.51 ± 2.91) were spread throughout the body, and the most extensive accumulation site 24 h after application was the spleen. Also, more significant accumulation was observed in tumor tissue and organs such as muscle, blood, heart, and brain. AuNTs alone do not cause a decrease in tumor tissue growth. The tumor volume tripled in 3 days was for RT (6 Gy) alone, while it was up to 8 days for the AuNTs + RT. The AuNTs + RT group had a significantly smaller mean tumor mass volume and improved mean survival than the RT group. Blood tests revealed no significant biochemical or hematological differences or histopathological changes in the kidneys, heart, and lungs. However, mice treated with AuNTs had foreign granuloma bodies, mononuclear infiltrates in the liver, as well, mononuclear infiltrates in the liver, and inflammatory infiltrates and granulomas in the spleen (50). Another tested shape was hollow AuNPs, and their radiosensitizing effect was tested in the breast cancer xenograft MDA-MB-231 by Mulgaonkar et al. (51). AuNPs (120 nm) showed from 5 to 6× accumulation tumor sites (97.4 ± 3.71% of gold was retained). After 30 days of treatment, hollowed AuNPs + RT (+10 Gy) showed more delayed tumor growth than RT alone. Several mice from the RT group had to be euthanized due to more aggressive tumor growth associated with their clinical conditions (dysfunctionality and limb swelling). At the beginning of the study, the RT group had a higher rate of cell death and necrosis causing visible scabs around the tumors. This caused insufficient blood flow, swelling, and loss of limb function. These conditions were not observed in the hollow AuNPs group. AuNPs alone have no therapeutic effect and also are not significantly toxic. The median survival of the AuNPs group was approximately 1.5× longer than that of the RT group (40 days) and 2.6× longer than that of other groups. Analysis of AuNPs’ biodistribution revealed the concentration of gold retained in tumors, and low concentrations were found in the liver and spleen, respectively (51). Chuang et al. (52) focused on the radiosensitizing effectiveness of gold nanodandelions (73 ± 10 nm) in mice bearing C6 glioma cells. The groups without RT were consistent in the rate and volume of tumor tissue throughout the experiment. This suggests that the gold nanodandelions alone showed almost no slowing tumor growth. RT (*D* = 16 Gy) slightly prevented tumor growth, and the combination of AuNPs + RT most prevented growth. Cytotoxicity was monitored among the groups. As there was not a single case of a significant decrease in body weight, it is clear that cytotoxicity did not occur (52).

In addition to the radiosensitizing effect, the higher atomic Z number of AuNPs also allowed combination with photothermal therapy (PTT). AuNPs with free electrons on the gold NPs’ surface can absorb electromagnetic energy of a specific wavelength (for example, emission by near-infrared [NIR] laser). Due to the interaction with NIR photons, AuNPs’ electrons become highly energized and release excess energy, heating the neighboring tissue. The resulting hyperthermia resulted in tissue damage, killing the tumor cells (53). In a study by Zhang et al. (54), they addressed enhancing the sensitization effect of AuNPs (40 nm) in combined RT and PTT within the MCF-7 model. Upon intratumoral injection of the AuNPs, the tumor’s positive photoacoustic and CT signals were enhanced 10 min after application, and the signal was stable for 2 h. AuNPs + PTT were irradiated with an NIR laser for 10 min, and after irradiation, the tumor tissue temperature increased by 13°C after 3 min; this was enough to inhibit the self-repair of damaged DNA. The combined therapy of AuNPs + RT had higher antitumor efficacy due to its improving inhibition of self-repair of damaged DNA, thereby slowing tumor growth. The combined therapy group of AuNPs +RT + PTT had the most significant level of apoptosis and necrosis production in cancer cells compared to the other groups. No animal in the experimental groups suffered a loss of body mass. Histological staining showed no toxicity or inflammation in vital organs (54).

A study by Li et al. (55) combined all the mentioned features, different shapes, and combined therapy. The radiosensitizing effects of 38 ± 2 nm nanobipyramids (NBPs) were evaluated by combining RT with PTT in mice bearing EMT-6 breast carcinoma. Regarding distribution, NBPs accumulated in the spleen and liver, the reticuloendothelial system organs, and, at moderate concentration, in the heart, lungs, and kidneys. This demonstrated the possibility of EPR accumulation in tumor tissues. For the study of NBPs as radiosensitizers, NBPs + RT (5 Gy) showed a better prevention effect, and NBPys + RT (8 Gy) had a milder inhibitory effect. No apparent changes in blood cell counts were detected, and no visible changes in hematoxylin–eosin (H&E) stained tissue. That means biosecurity and biocompatibility. The study further focused on synergistic combined RT with NBPs and PTT. In mice irradiated with laser, PTT had a negligible tumor reduction effect. NBPys + RT and NBPys + PTT had similar impacts. The combination of NBPs + PTT and subsequent RT enhanced the reduction effect. The most significant effect was in the group wherein NBPs were combined with RT and PTT used before and after the radiation. This combined therapy caused the elevation of hypoxia in tumor tissue which affected DNA repair in the tumor (55).

### 3.2 Surface-modified AuNPs

Due to their surface area, AuNPs can easily be functionalized using various ligands, polymers, and biomolecules. The molecules that were attached to the surface of AuNPs comprise a relatively heterogeneous group, including groups of different carbohydrates or drugs to improve the result of the therapeutic effect, natural coupons; various peptides; signaling molecules; hormones; monoclonal antibodies for more targeted and specific delivery to the tumor site, or influencing the tumor microenvironment; or substances to support the radiosensitization process (Table 2).

The earliest research has used a combination of already known anticancer molecules, binding them onto the surface of AuNPs by EPR to improve tumor accumulation and thereby obtain better therapeutic results. An example of this approach is seen in a study by Chattopadhyay et al. (56), where they aimed to enhance the radiation effects of AuNPs (*d* = 30 nm) coated with trastuzumab (AuNPs-T) in MDA-MB-361 xenograft in mice. Trastuzumab is a humanized monoclonal antibody that interferes with the HER2 receptor. Overexpression of HER2 promotes invasion, survival, and angiogenesis of tumoral cells. It is mostly used in breast or stomach cancer. Based on experiments with various IR doses (0, 2, 6, 11, or 15 Gy), a dose of 11 Gy was selected to evaluate the radiosensitizing properties of AuNPs. Analysis of tumor regression showed AuNPs-T + RT to result in slower tumor growth compared to the RT group. During 4 months, survival analysis showed no differences. This indicates no toxicity in normal tissue due to using AuNPs-T + RT (11 Gy). The blood count and serum levels measured did not show significant differences except for creatinine levels in the RT group (56). Similar approaches have been used in studies by testing AuNPs + cisplatin (57) or gold nanorods and fluorescent NPs loaded with doxorubicin (DOX) (56). Cisplatin and DOX are commonly used chemotherapeutic drugs with anticancer activity in various tumors. Both drugs interfere with DNA repair mechanisms, causing DNA damage. Zhang et al. (57) in their study used a system of pH-responsive self-aggregating AuNPs + cisplatin (75.02 nm) or added a hydrophilic molecule, peptides 2,3-dimethyl maleic anhydride (DMMA) (78.3 nm) in mice bearing melanoma. Gold retention after 24 h was significantly greater in the group treated with AuNPs + cisplatin + DMMA (15.75% ± 1.01%) compared to other groups. Also, this group saw the greatest effect in inhibiting tumor growth even without RT (4 Gy). Combination with RT had the best antitumor effect and inhibition of tumor growth. The treatment caused necrosis but did not cause side effects (57). Analogous results were observed by Chen et al. (58) using AuNPs with DOX (122 nm) in mice bearing MCF-7. The fluorescent probe used lanthanide-doped down conversion nanoparticles (DCNPs) for guided RT and pH-responsive vesicles as carriers for DOX for improved resealed of therapeutic. During imaging of the applied AuNPs, robust signal detection was seen in the tumor area, while the signal was only low in the other tissues. Modifying the DOX into a pH-responsive vesicle increased the positive signal in the tumor up to 48 h. These results show that irradiated (5 and 10 Gy) DOX-loaded pH-reactive vesicles have significant radiosensitization due to the combined chemoradiotherapy. H&E staining showed little toxicity to vital organs after 18 days, and the tumor tissue showed slight necrosis in the group with applied AuNPs. The combination of DOX-loaded pH-responsive AuNPs + RT was very effective in inducing massive apoptosis of tumor cells (58). Not only anticancer drugs are attached to AuNPs surface. For example, Shi et al. (59) used low doses of tiopronin-coated ultrasmall AuNPs (2.77 ± 0.69 nm) in mice bearing human colorectal carcinoma. Tiopronin (Tio) is a low-molecular thiol derivative of glycine used to treat various conditions. It is mainly used in tumor diseases due to its antioxidant properties and ability to suppress side effects. Compared to the untreated group, Tio-AuNPs were not toxic and did not adversely affect tumor growth when administered i.v. or i.t. The i.t. Tio-AuNPs + RT (10 Gy) resulted in significant radiosensitization of tumor tissue compared to the IR group or i.v. Tio-AuNPs + RT (10 Gy) (59). In a study by Jia et al. (60), they addressed the radiosensitizing effects of levonorgestrel-coated gold AuNP clusters (Au_8_, 2 nm) in EC1-bearing mice. Levonorgestrel (LEV) is a synthetic progestogen, a first-line oral contraceptive known as a morning-after pill. It also is used in intrauterine birth control systems. Biodistribution has shown the greatest accumulation in tumors but low accumulation in mouse organs except for the bladder and without detected abnormalities in the organs. The examined tumor showed LEV-AuNPs + RT (4 Gy) to reduce tumor growth, and the radiosensitizing effect was confirmed (60). In a study by Wang et al. (61), they addressed the radiosensitizing effects of 8-hydroxyquinoline (8-HQ)-coated AuNPs (58 nm) in CD31-bearing mice. 8-HQ is a compound derived from the heterocycle quinoline and metal chelator with antiproliferative activity. 8-HQ has been used in designing various classes of anticancer compounds through a synergistic effect on anticancer drugs by altering their pharmacokinetic characteristics and improving their therapeutic potential. The 8-HQ-AuNPs showed changes in tumor vessel morphology, increasing perfusion and improving tumor hypoxia. The 8-HQ-AuNPs + RT (4 Gy) showed the highest tumor inhibition rate and caused the greatest tumor necrosis, cell change, and nuclear condensation (61).

Due to their rapid proliferation and expansion, cancer cells are demanding energy (glucose). Studies have shown glucose-capped AuNPs to have improved intake by target cancer cells. These approaches were used in the study by Li et al. (62), who dealt with the radiosensitizing effects of combining AuNPs with surface-bound glucose and applied curcumin in mice with transplanted breast cancer MDA-MB231-luc. Curcumin is an herbal compound of the turmeric plant that has proven anticancer effects through various mechanisms (modulation of antiapoptotic genes, activation of caspases, reducing modification of matrix metalloproteases, among others). The group with cisplatin showed the slowest growth and smallest tumor volume. After IR (10 Gy), the tumor volumes decreased significantly, especially in those groups that received the combination of curcumin with AuNPs or curcumin alone. The intensity of bioluminescence–luciferase on AuNPs was used to detect tumor accumulation. The group with applied curcumin + Glu-AuNPs+ RT had the highest tumor accumulation. H&E staining showed large and heterogeneous nuclei with minimal cytoplasm and central necrosis in the tumor (62). On the contrary, Jia et al. (63) were devoted to the enhanced effects of AuNP clusters (2.59 nm) coated with D-fructose in mice bearing a HeLa tumor. The tumor volume in the group treated with D-fructose and AuNPs + RT (4 Gy) was significantly lower. No changes in body weight or pathological damage to the tissues of vital organs were recorded (63).

Polysaccharides, too, are used intensively as modifying agents on the AuNPs surface. Hyaluronic acid or chitosan molecules are frequently used due to their biological effects and improved tumor penetration and retention. In a study by Xu et al. (64), they addressed the nanotheranostics effect of hyaluronic acid-coated gold nanocages (HA-AuNPs, 193.3 ± 8.1 nm) in mice bearing 4 T1 breast carcinoma. The greatest accumulation of HA-AuNPs was recorded at 24 h and gradually decreased after 2 days. Moreover, HA-AuNPs-treated tumors contained much more gold. At 21 days after injection, the group HA-AuNPs + PTT + RT (6 Gy) showed strong arrest of tumor growth (64). Chitosan was used in the study by Hua et al. (65), where they worked with multilevel responsive clustering of AuNP nanoclusters coated with fluorescent green dye and chitosan (Cs-AuNPs-ICG; 50 nm). They observed enhanced synergistic RT (11 Gy) and PTT in mice bearing 4 T1 metastatic breast cancer. Fluorescence revealed a greater accumulation of Cs-AuNPs-ICG in the tumor even after 72 h. They furthermore investigated the synergistic antitumor effect of Cs-AuNPs-ICG with PTT. In the group with Cs-AuNPs-ICG, the temperature of the tumor rose rapidly to as high as 54°C while the control rose only to 41°C. Subsequently, they evaluated the antitumor effect. Both PTT and RT induced the radiosensitization effect. The Cs-AuNPs-ICG group showed better tumor inhibition. The group treated with Cs-AuNPs-ICG + PTT + RT had the most optimal effect on tumor inhibition; however, in contrast with other groups, metastatic lesions did not appear in the lungs and liver. The histological staining of Cs-AuNPs-ICG revealed minimal toxicity (65).

For more active targeting delivery to tumors, one of the strategies is to modify the AuNPs’ surface with peptides, hormones, antibodies, proteins, or various molecules with biological properties already proven to enhance their biocompatibility, uptake, and biological effect on tumors. Among the examples are well-known peptides such as glutathione (66, 67) or integrins interaction-blocking peptides such as arginine-glycine-aspartic acid (cRGD) or longer 9-amino acid iRGD (68, 69) or arginine-alanine-aspartic acid (cRAD) (68). Glutathione (GTH) is a naturally occurring thiol peptide with a central role in ROS regulation and antioxidant tissue systems, and these were used in studies by Zhang et al. (66, 67). Both studies dealt with the radiosensitizing effects of ultrasmall AuNPs. These nanoclusters contain only 10–12 (66) or 29–43 atoms of (67) Au, and thus smaller than 3 nm, in mice bearing the U14 tumor. The tumor uptake of GTH-Au_29-43_NPs was lower than that of GTH-Au_10-12_NPs. Both types of AuNPs were located in key organs, including the kidney, liver, spleen, heart, and lung at 24 h. Tumor volumes and blood tests were measured for radiosensitization effects. The greatest decrease was recorded in both groups treated with either GTH-Au_10-12_NPs + RT (5 Gy) or GTH-Au_29-43_NPs + RT (5 Gy). Although GTH-Au_10-12_NPs treatment had reduced blood chemical and biochemical values with 23-day recovery, GTH-Au_29-43_NPs showed no significant weight loss or changes in organs or blood counts (66, 67). Contrary to GTH NPs, in another study by Zhang et al. (70), they designed AuNP with histidine, which could bind tightly and selectively with intracellular GSH in U14-bearing mice. As a comparison group, H-AuNPs-GSH were synthesized. Both AuNPs without RT have no radiosensitizing effect. H-AuNPs + RT (6 Gy) have an excellent radiosensitizing effect, as detected by tumor growth inhibition, and no apparent systemic toxicity in the organs, including heart, liver, spleen, lung, and kidneys was observed, thereby indicating excellent biocompatibility (70). In a study by Liang et al. (68), they compared the radiosensitization of fluorescent AuNPs modified with cRGD or modified cRAD peptides in mice bearing 4 T1 breast cancer. CT imaging signal, tumor uptake, and accumulation were more intensive and stable for cRGD-AuNPs than cRAD-AuNPs. Only cRGD-AuNPs were used to test the effect on radiosensitization. Their elimination half-life was 124.3 min. Compared to other groups, antitumor efficacy was presented mainly in the combination cRGD-AuNPs + RT (6 Gy). At the same time, they did not show organ toxicity (68). In a study by Dong et al. (69), they compared the radiosensitizing effects of AuNPs (5.2 nm) and iRGD-AuNPs additionally modified with *α*-difluoromethylornithine (10.6 nm), which can be released from AuNPs to the tumor environment and regulate the cell cycle of tumor cells. AuNPs were tested in GL261 glioma-bearing mice. The group with iRGD-AuNPs showed higher selectivity and accumulation in glioma due to more effective blood–brain barrier penetration. Irradiation (4 or 8 Gy) did not affect the accumulation of iRGD-AuNPs in gliomas. Also, the iRGD-AuNPs group achieved excellent tumor inhibition efficacy at doses of 8 Gy. This phenomenon confirmed the RT sensitization of iRGD-AuNPs in gliomas (69). Prostate membrane specific antigen (PSMA-1) peptide was used in a study by Luo et al. (71) on ultrasmall AuNPs (3.0 ± 0.7) nm or PSMA-1-AuNPs (5 nm) in mice bearing prostate cancers (PC3pip and PC3flu tumors). Successful accumulation of PSMA-1-AuNPs in the tumor was confirmed, with a peak at 4 h and elimination within 24 h. The group of PSMA-1-AuNPs + RT (6 Gy) mice showed a reduced rate of tumor growth and increased weight of mice. The authors concluded that PSMA-1-AuNPs enhanced the radiosensitizing effect more in PC3flu compared to PC3pip tumors (71). In a study by Nicol et al. (72), they looked at the radiosensitizing effects of double peptide sequence-coated RME and H5WYG AuNPs (36.6 ± 3.07 nm) in mice bearing MDA-MB-231 breast cancer. The biological effect of RME and H5WYG in promoting internalization and destabilizing the endosomal membrane is a process known as the proton sponge effect. Single peptide RME-AuNPs (28.7 nm ± 0.89 nm) and H5WYG-AuNPs (45.9 ± 0.92 nm) were tested *in vitro*. Modified AuNPs with both peptides without RT did not affect tumor growth. On the contrary, AuNPs + RT (4 Gy) significantly delayed tumor growth and had the highest survival (72). In a study by Ma et al. (73), they addressed the radiosensitizing effects of AuNPs in the shape of nanospikes (114 ± 40 nm) coated with a transactivator of transcription (TAT) cell-penetrating peptide in mice bearing U14. For the biodistribution TAT-AuNPs were mainly spread to the liver and spleen. The sensitization effect of TAT-AuNPs + RT (6 Gy) showed significant tumor destruction and upregulation of autophagy. This study also tested AuNPs coated with folic acid, but tested only *in vitro* conditions (73).

Apart from peptides, proteins with different biological functions can be attached to the AuNPs surface. We have found that antibodies and hormones are frequently used. In a study by Liu et al. (74), they addressed the radiosensitizing effects of functionalized AuNPs (2.6 nm) coated with Rho antibody (16.3 nm) in mice bearing ER+, HER-2−, and PR− xenograft. RhoJ endothelial-expressed is a protein involved in tumor angiogenic and vascular integrity. Recently, the connection between RhoJ regulation of epithelial-to-mesenchymal transition has been confirmed. Also, this study compared the effect of RhoJ-AuNPs with bevacizumab monoclonal antibody against vascular endothelial growth factor A. The tumor volume of the RhoJ-AuNPs + RT (5 Gy) decreased for the first 6 days, and subsequently regressed and reached complete elimination on the 20th day. The treatment of RT, bevacizumab, or RhoJ-AuNPs did not completely inhibit tumor growth. RhoJ-AuNPs + RT affects receptors for vascular endothelial growth factor and platelets by inhibiting angiogenesis, thereby protecting against tumor recurrence. Results confirmed the antitumor and radiosensitizing effects of RhoJ-AuNPs + RT, without abnormalities or inflammatory lesions, and caused low toxic effect on the liver, kidney, and spleen (74). In a study by Gal et al. (75), they tested the radiosensitization of insulin-coated AuNPs and cetuximab antibodies (CTX-INS-AuNPs) in mice bearing human glioblastoma U-87 MG. Cetuximab is an epidermal growth factor receptor inhibitor that blocks phosphorylation, resulting in the downstream of multiple important signaling pathways downstream. This study also compares/combines the efficiency of AuNPs vs. temozolomide, a molecule used in treatment of the glioblastoma. A brain CT scan showed CTX-INS-AuNPs have 15× greater uptake than the free antibodies. The combined treatment of CTX-INS-AuNPs + temozolomide + RT (10 Gy) caused a significant inhibition of tumor tissue growth. However, all groups were confirmed tumor cells in the brains by H&E staining, but combined treatment destroyed epidermal growth factor receptors (75). The study by Wolfe et al. used coated AuNPs (100 nm) with goserelin, a synthetic analog of gonadotrophin hormone in Foxn1^−/−^ mice bearing prostate cancer. The treatment with goserelin-conjugated AuNPs + RT (5 Gy) delayed tumor growth, contrary to only PEG-conjugated AuNPs + RT when this effect was not observed (76). In a study by Ghahremani et al. (77), they used AuNPs (7.7 nm) and AuNPs coated with tumor cell growth-reducing protein (AS1411 aptamer) (15.2 nm) in mice bearing 4 T1 breast cancer. They investigated tumor inhibition for the radiosensitizing effect. AuNPs or AS1411-AuNPs are safe and did not cause organ toxicity AS1411-AuNPs + RT (6 Gy) significantly slower the tumor growth compared to AuNPs + RT where the radiosensitizing effect was not observed (77).

The very common substance modified to AuNPs surface is folic acid (FA). FA is a well-known vitamin important in nucleotide biosynthesis and biological methylations. Due to this role in organisms, the impact of FA on cancer development is intensively studied but remains controversial. In a study by Kefayat et al. (78), they addressed the radiosensitizing effects of ultrasmall AuNPs (5.5 ± 0.4) coated with FA in glioma-bearing Wistar rats (78). FA-AuNPs biodistribution revealed higher accumulation in tumors than in brain tissue and confirmed biological safety. FA-AuNPs+RT (6 Gy) prolonged the survival time and improved tumor radiosensitization. FA-AuNPs without RT significantly reduced the Ki-67 tumor proliferation marker but did not affect survival (78). In a study by Cheng et al. (79), they designed AuNPs coated with FA (23 ± 1.6 nm) and/or added photolabile diazirine group (DA) suitable for 405-nm laser irradiation in 4 T1-bearing mice. Results of the radiosensitization efficacy on tumor volume and weight showed the highest inhibition in FA-AuNPs-DA + RT (2 × 4 Gy) + 405 nm laser (prior IR), and in this group, any lung metastases were observed (79). Further study by Ding et al. (80) designed AuNPs coated with sulfenic acid reactive groups 1,3-cyclohexanedione (CHD), reducing exocytosis of gold in tumors and/or FA (25 ± 3.2 nm) in 4 T1-bearing mice. Also, the results of this study confirmed that the best radiosensitization effect on tumor volume and weight were observed in the group applied with FA-AuNPs-CDH + RT (8 Gy), which suggests the excellent radiosensitizing efficacy of FA-AuNPs-CDHin cancer radiotherapy (80).

Furthermore, another option to coat the AuNPs surface is fragments of RNA or small interfering RNA (siRNA) to silence/upregulate gene expressions. AuNPs are intensively used as a scaffold for effective and safe gene delivery *in vivo* application. This approach has been used in studies by Masood et al. (81) and Yang et al. (82). In the first study, AuNP nanorods (50–70 nm) were coated with siRNA against SphK1 (sphingosine kinase 1 gene) in a human squamous cell carcinoma xenograft. The mice showed no signs of toxicity, and body weight remained normal. The treatment with SphK1-AuNPs with or without RT (1.0 Gy twice a week up to 25 days) reduced tumor volume. These results were not observed in groups treated with AuNPs or non-specific siRNA-AuNPs. Furthermore, the antitumor effects and efficiency to downregulate SphK1 expression were evaluated. The SphK1-AuNPs reduced the expression of the gene but without a significant difference with/without RT; also, this treatment significantly reduced the percentage of proliferating cells in the tumor and increased apoptosis, which supports the radiosensitization with siRNA delivery technology (81). The second study addressed the radiosensitizing effect of gold AuNPs in dendrimer polyplexes (175.7–206.7 nm) coated with siRNA for HIF-1α, as a key regulation protein to induce RT resistance in A549-bearing mice. For radiosensitizing effects, HIF-1α-AuNPs + RT (6 Gy) and siRNA-AuNPs + RT showed greater tumor inhibition effects compared to the other group, nevertheless, HIF-1α-AuNPs + RT had the smallest tumor size, the highest level of tumor necrosis and cell nuclear breakdown and the highest level of *γ*-H2AX. No visible changes in body weight were detected. The gold accumulation gradually increases in the kidneys and is excreted in the urine within 48 h. Moreover, HIF-1α silencing may result in downstream angiogenesis, invasion, and metastasis of tumors. Results have shown that application of HIF-1α-AuNPs with and without RT significantly downregulated HIF-1α protein expression, matrix metalloproteinase 9 (invasion), and vascular endothelial growth factor (angiogenesis and metastasis), and H&E staining revealed reduced metastatic lesion in lung tissue (82).

### 3.3 Combined AuNPs with different atoms

The unique physical and chemical properties of AuNPs are well-known. However, the combination of two or more elements the NPs made resulted in various more comprehensive applications and significant improvements in diagnosis and therapy. These complex multifunctional systems can still be surface modified with different molecules, allowing almost unlimited applicable potential (Table 3).

Non-metal silica nanoparticles (SiNPs), composed of silicon dioxide, are mainly used due to excellent stability, porosity, non-toxicity, biocompatibility, easy surface modification, excellent dispersion, and high thermal resistance. The physical properties of SiNPs included great adsorption capacity of energy. Current applications involved mainly nano-carriers for drug delivery and biosensors. In the study by Zhao et al. (83), they used a combination of AuNPs and Si in nanodots with a surface modification with cRGD peptide (57.70 nm) in MDA-MB-231 xenograft-bearing mice. Accumulation of cRGD-SiAuNPs was observed in the heart, liver, lung, kidney, and spleen, however, retention of the modified NPs in the tumor was up to three times longer than untargeted SiAuNPs. Tumor growth was significantly delayed in the cRGD-SiAuNPs + RT (10 Gy) with the highest tumor growth reduction (83). In a study by Chiang et al. (84), they used SiAuNPs modified on the surface of HA (187.1 ± 3.3 nm) and combination delivery with 5-aminolevulinic acid (sonosensitizer) for more targeted uptake in the tumor site in glioblastoma multiforme murine model. Biodistribution was determined in both mouse and rat model Fisher 344. Toxicological, histological, and biodistribution analysis and hematological results did not show serious damage to vital organs. To determine the effect of radiosensitization, eliminated glioblastoma cells were observed in combined therapy of HA-SiAuNP +5-aminolevulinic acid + application of ultrasound + RT (2 Gy) and prolonged survival median time compared to other groups (84). A new study by Wang et al. (85), employed a triple of radio-, immuno- and antiangiogenic therapy—“therapy trident” with SiAuNPs (143 nm) subsequently surface modified with the substances toripalimab (a monoclonal antibody against programmed death protein 1) and bevacizumab, either individually or in a combination of both substances in Huh-7 mice-bearing xenografts. An improved sensitization effect on tumor growth was confirmed by a combination of toripalimab + bevacizumab-SiAuNPs + RT (5Gy). A combination of toripalimab + bevacizumab as conventional therapy and toripalimab + bevacizumab-SiAuNPs without RT had a similar effect and suppressed tumor growth. Biocompatibility and non-toxicity of this therapy were confirmed by H&E staining (85).

Another common combination is AuNPs with another metal element, iron (Fe). This common atom is widely prevalent in the form of iron oxide in minerals such as hematite, maghemite, goethite, and magnetite. The unique properties of FeNPs, such as their superparamagnetism (SPIONs aforementioned), ability to induce hyperthermia for tumor treatment (through magnetic or PTT methods), and biocompatibility, make these nanoparticles increasingly valuable not only for diagnostic and imaging purposes but also for drug delivery and cell tracking systems. Moreover, FeNPs revealed their role in immunotherapy due to their interaction with the immune system, which stimulates tumor recognition and enhances cancer therapy (86). Their combination of Fe with Au atoms has been used in the study by McQuade et al. (87), where they used SPION and AuNPs in micellar complex (100 nm) on the model of fibrosarcoma. FeAuNPs tolerance was good, with no observable behavioral changes or hepatic or gastrointestinal signs. The circulating clearance half-life of FeAuNPs was 1.45 h for the distribution phase and 17.5 h for the elimination phase. FeAuNPs + RT (6 Gy) showed a significant improvement in survival and complete response without any detectable tumor (87). In a study by Chen et al. (88), they applied FeAuNPs (100 nm) without surface modification but in combined therapy with PTT in mice bearing 4 T1 tumor. The combined therapy of FeAuNPs + PTT + RT (4 Gy) had the highest tumor growth reduction efficiency. At the same time, this therapy had a higher mortality of tumor cells compared to the control groups (88). In a study by Nosrati et al. (89), they used complexes of surface-modified FeAuNPs with FA and curcumin (138.4 nm) in mice bearing 4 T1 tumors. The combined therapy of curminine + FeAuNPs-FA + RT (2 Gy) was the most effective group that eradicated the tumor and was the only one without any death. The biocompatibility and toxicity of FeAuNPs-FA revealed no changes or adverse effects (89). The next study by Hua et al. (90) involved modifying the surface of the AuNPs (size as Au_4_ cluster) in combined FeAuNPs using cRGD and testing the FeAuNPs in 4T1 tumor-bearing mice. The cRGD-FeAuNPs + RT (4 Gy) group showed a degree of reduced tumor volume and weight compared to the other groups. The study confirmed targeted tumor delivery based on cRDG presence and the radiosensitizing effect of FeAuNPs (90).

Another non-metal atom used to combine with AuNPs is selenium (Se). Se compounds have importer regulation and ROS catalytic role in the organism, but it was shown that SeNPs have lower toxicity and higher biocompatibility than organic or inorganic Se compounds. Due to their physical properties, surface charge, and hydrophobicity are intensively used in therapeutic applications. In a study by Chang et al. (91), they involved the combination of AuNPs and Se in the form of nanorods (120 nm) in mice with a melanoma xenograft. The i.t. application of SeAuNPs + RT (4 Gy) demonstrated higher therapeutic efficacy than i.v. Biodistribution analysis showed that SeAuNPs mainly accumulate in the liver, spleen, and tumor and showed no toxic signs of damage or inflammation. Both groups with SeAuNPs + RT showed significant tumor damage and irregular expansion of the intercellular space (91). Another study used a combination of three atoms to construct NPs: Au, Se, and copper (Cu) in nanocrystals (17.6 ± 1.4 nm) in mice (92). In this study Cu is a transition metal with interesting physical and chemical properties (mechanical, electrical, and thermal), but also shown significant antibacterial effects. In the study by Huang et al. (92), they tested CuSeAuNPs radiosensitization and the PTT effect in mice bearing 4 T1 tumor cells. Toxicity indicates that small doses of CuSeAuNPs do not cause serious health problems and that their effects on the immune system (decreased white blood cells and platelets) are mild and temporary. CuSeAuNPs were generally found in the liver and spleen without serious damage. Tumors from mice treated with CuSeAuNPs + RT (6Gy) + PTT were successfully ablated without relapse. The results showed 100% survival of the mice with combined therapy. Mice from all groups except CuSeAuNPs + RT + PTT developed severe liver metastases that proved tumor radiosensitization (92).

Another very common dualling atom is manganese (Mn) from manganese oxide. MnNPs have very good biocompatibility and catalytic activity and are often used in MRI imaging as enhanced contrasting agents due to bright signals. In a study by Yi et al. (93), they tested the efficacy of radiosensitization of MnAuNPs (100 nm) compared to MnNPs or AuNPs in 4 T1 tumor-bearing mice. Toxicity of MnAuNPs did not reveal any observable organ damage or inflammation. A hypoxia study revealed that MnNPs and MnAuNPs significantly reduced the hypoxic signals in the tumor. At the same time, AuNPs did not affect hypoxia. Conversely, MnAuNPs + RT (6 Gy) significantly suppressed tumor growth and the highest level of cell apoptosis compared to groups with single-atom NPs (93). The study by Chen et al. (94) investigated the enhanced theranostic effect of MnAuNPs with BSA for improved biostability in mice bearing 4 T1 tumors. Similar results with hypoxia as the previous study were recorded, profiting MnAuNPs. The radiosensitization study revealed that MnAuNPs + RT (6 Gy) appeared to be the most effective in tumor inhibition. The combination of properties Mn (antioxidation of H_2_O_2_) and Au (enhanced tumor damage based on IR energy deposition) is very suitable for cancer treatment (94). In a study by Lin et al. (95), they composed Janus MnAuNPs modified with GSH to investigate synergistic effects with combined PTT in MCF-7 tumor-bearing mice. Applying GSH-MnAuNPs did not cause significant changes in weight or pathological signs of vital organs, indicating no systemic or chronic toxicity. To assess the antitumor sensitizing effect, application of GSH-MnAuNPs + RT (4 and 6 Gy) inhibited the growth of tumors and caused extensive necrosis, continuously of GSH-MnAuNPs are suitable contrasting agents for multimodal imaging therapy (95). The following study combined three atoms of Au, Mn, and zinc (Zn) in mice bearing 4 T1 tumors. ZnNPs are metallic and have advanced non-toxic, antimicrobial, antibacterial, and excellent UV-blocking properties. This study used the Zn shell to stabilize and protect the Mn shell from oxidization in aqueous solutions. ZnMnAuNPs (30–40 nm) passively accumulate in tumors, most likely due to the EPR effect with prolonged blood circulation. Biodistribution showed a high accumulation of NPs in the RES, including the liver and spleen, but no significant side effects were observed in organs or inflammation. A combination of ZnMnAuNPs + RT (4 Gy or 6 Gy) inhibited tumor growth and caused severe DNA damage, but this effect wasn’t observed without RT, which suggests the radiosensitizing effect of ZnMnAuNPs (96).

Other metal atoms that were used to combine with AuNPs were platinum (Pt) (97), titanium (Ti) (98), or palladium (Pd) (99, 100). PtNPs are noble metals with distinctive physiochemical properties (photothermic, high surface area, and catalytic), however, studies of their toxicity are quite contradictory about the safety of the application. In a study by Yang et al. (97), they designed PtAuNPs (150 ± 20 nm), combining radiosensitizing and catalase-like properties (nanozyme) of atoms in mice bearing 4 T1 tumors. The concentration of PtAuNPs was detected in tumors (EPR effect of Au) and in several organs, mainly the liver, when RES. The radiosensitizing effect was confirmed based on tumor inhibition in PtAuNPs + RT (8 Gy), and H&E staining revealed significant morphological changes and apoptosis of cancer cells, but no side effects on vital organs (97). In a study by Cheng et al. (98), they composed TiAuNPs (79.6 ± 5.4 nm) in SUM159-tumor-bearing mice. The combination of Ti and Au act synergically due to asymmetric electric coupling between atoms. Due to the EPR effect, TiAuNPs accumulated in the tumor, however, the highest amounts were in the liver and spleen. Tumor growth was significantly reduced in the TiAuNPs + RT (10 Gy) with the longest survival. Neither H&E staining nor histological examination revealed damage to vital organs (98). Further studies combined AuNPs with the noble metal Pd. PdNPs’ properties include ROS production activity, DNA damage, and PTT agents with potential in antitumor therapies. Liu et al. (99) designed PdAuNPs with different compositions of Pd and Au atoms. The composition Pd_20_Au_80_NPs (10.43 ± 1.55) displayed the highest catalytic activity on ascorbate oxidation and was subsequently chosen for radiosensitization of 4 T1 tumor-bearing mice. The distribution of PdAuNPs (with PEG) showed accumulation in the tumor and liver, with minimal liver toxicity and no renal toxicity. The highest radiosensitizing effect by tumor inhibition was reached by a combination of ascorbate (i.p. prior RT) + PdAuNPs + RT (4 Gy) observed with the highest level of tumor necrosis. Interestingly, ascorbate + RT and PdAuNPs + RT also exhibited a lower radiosensitizing effect (99). Also, Xiang et al. (100) looked at the radiation-enhancing effects of PdAuNPs (*d* = 50 nm) for combined RT and PTT in mice bearing colon cancer MC-38 cells. For better biostability and tolerability, polyvinyl pyrrolidone (PVP) was added to the surface of PdAuNPs. Double combination with PTT agents demonstrated a remarkable degree of heating, temperatures exceeded the photoablation limit of 50°C. Combining PdAuNPs + RT (2 × 5 Gy) and PdAuNPs + PTT showed significant tumor inhibition, however, PdAuNPs +PTT + RT caused extreme tumor clearance (100).

## 4 Discussion

Advancements in radiation therapy have taken significant strides over the past decade at a time when NPs have been emerging as versatile tools across various fields. In particular, NPs have gained substantial recognition in the realm of medicine. This review addresses the radiosensitization effects of AuNPs in experimental *in vivo* studies. Our collected data highlight that AuNPs are very well tolerated and do not cause significant toxicity. Only a few studies have described significant signs of toxicity as indicated by changes in body weight or organ infiltrations. There is no direct link between these studies, however, because body weight loss was observed in applying unmodified 4 nm spherical NPs (49) or after surface modification with 36.6 nm RME and H5WYG peptides AuNPs (72) and for 54.9 nm TAT peptide AuNPs (73). A study of biodistribution and evaluation of organ toxicity indicates that 61.51 nm nanotriangles caused infiltration of the liver and spleen (50), modified 53.1 nm chitosan nanospikes were reported to have minimal toxicity to the liver and lung (65), and anti-RhoJ 16.3 nm nanoclusters affected liver, kidney, spleen, and inflammation markers (74). Biodistribution studies showed that the method of NPapplication significantly affects the outcome. Greater radiosensitization was achieved with intratumoral (i.t.) application. After i.t application, AuNPs maximize their accumulation at the tumor site compared to other application routes. A higher concentration of gold in tumors caused more damage and enhanced radiosensitizing effect due to gold’s direct absorption of X-rays (59). Otherwise, biodistribution and pharmacokinetics based on the application route vary greatly across the literature. One of the major factors in this regard is the size and modification of AuNPs, although all AuNPs are predominantly taken up by tumor tissue. Moreover, smaller particles (<6 nm) can avoid significant liver uptake and be found in muscles (1.9 nm, unmodified (41)) or kidney and liver (3.2 nm modified with cRAD and cRGD peptides (68) or 5.5 nm modified with FA (78)) after i.v. application and in the kidney after intraperitoneal injection (2 nm, modified with levonorgestrel (60) or GTH (66, 67)). Also, kidney uptake can be avoided by surface modification (such as by PSMA-1 peptide), which is preferable in prostate cancer therapy (71). A consistent trend was seen in the cases of “bigger” NPs (<10 nm), thus confirming biokinetics through the kidney and spleen, and additionally the lungs, after i.v. application (45, 46, 50, 51, 76, 87). Additionally, shreds of scientific evidence point to greater survival of tumor-bearing mouse models treated with AuNPs + RT in contrast to radiation-only or AuNPs groups. Multiple studies confirmed a > 80% survival rate in AuNPs + RT groups (41, 45, 49, 51, 77, 78). On the contrary, our intensive literature search to prepare this review has shown the weakness of NP research. Although evidence of the radiosensitization effects of gold NPs is undeniably confirmed through experimental *in vivo* studies, the experimental setups are piecemeal (reporting incomplete information on elimination times, pharmacokinetics, various time intervals of biodistribution, survival analysis, and organ toxicity) and nonuniform. A standardized protocol with a few specific “must include” experimental analyses is needed for further studies. This approach could markedly close the missing data gaps, thereby contributing to a fuller understanding of AuNP efficiency in tumor therapy.

We summarized the radiosensitizing potential of non-modified, modified, and combined AuNPs. All studies confirmed that the application of AuNPs without RT has minimal or no effect on tumor growth. However, when used with ionizing radiation or PTT, AuNPs of every size, shape, and modification have a significant radiosensitizing effect. Similar findings confirmed that unmodified or modified NPs combined with X-ray resulted in maximal reduction of tumor weight that is consistent with smaller tumor volume and greater growth inhibition, and even in total ablation or elimination. Subsequently, some of the publications cited herein delved deeper into the different effects of AuNP. The induction of apoptosis and occurrence of apoptotic tumor cells for radiosensitization was explored. It was confirmed that the combination therapy with AuNPs + RT had the strongest impact on apoptosis in tumor cells without inducing side effects in the organism. Substantially, this group has a sharper increase in apoptotic signals compared to the radiation-alone group (45, 47, 61, 75, 78, 80). A study comparing the effect of different AuNP sizes (187, 50, and 8 nm) determined size-dependent induction of apoptosis genes favoring small and medium-sized NPs (48). Approaches with high treatment efficiency combine AuNPs’ properties with other antitumor therapies. Even so, AuNPs accumulate specifically in cancerous tissue while sparing normal tissues, and this effect can be amplified with surface modification. Combined AuNPs + RT with chemotherapies options are proving favorable results. All studies incorporating chemotherapeutic agents (e.g., trastuzumab, cisplatin, doxorubicin, cetuximab, toripalimab, bevacizumab, etc.) on the surface of NPs have proven the great combined effect of this approach as seen in a significant reduction in tumor volume compared to control or RT alone groups (56–58, 85). Remarkable success has been achieved when AuNPs are combined with RT together with PTT. Radiosensitive efficiency can be enhanced by inhibiting the self-repair of damaged DNA, thereby intensifying the tumor growth. The antitumor efficiency of combination therapy is achieved through an interplay of three influences: relieving tumor hypoxia by dilating tumor blood vessels, concentrating higher radiation energy locally within the tumor, and NIR laser inhibiting the repair of radiation-induced DNA damage (54, 55, 64). On top of that, in combination with different atoms, NPs can acquire a new property; for example, catalytic (Mn or Pd) or a better option for multimodal imaging (MRI, CT, ultrasound, or photoacoustic). FeAuNPs + RT demonstrated a positive photothermal effect. The gold core acted as a radiosensitizer, triggering X-ray deposition within tumor cells to induce cell apoptosis. At the same time, the FeS shell offered contrast for both T2-weighted MR imaging and photoacoustic imaging, as well as high NIR absorbance to enable PTT (88). Another positive effect of PTT resulting in extreme tumor ablation was demonstrated by PdAuNPs +PTT + RT (100) and CuSeAuNPs + RT (6Gy) + PTT (92). Demonstrated coactive effect and almost unlimited options for surface modifications, AuNP brings brighter possibilities for improved tumor targeting, accumulation, and enhancing therapeutical results.

AuNPs were often used in various breast carcinoma models (MCF-7 tumor or MDA-MB-361 tumor cells). Studies have demonstrated the favorable permeability of AuNPs into the tumor, both for the NPs themselves and their surface-modified counterparts. Interestingly, the size range of AuNPs varied from 1.9 ± 0.1 nm (42) to 193.3 nm ± 8.1 nm with HA (62). The study involving the smallest AuNPs (spherical 1.9 ± 0.1 nm, i.v.) indicated that the tumor growth outcome depends on the dose of i.v. application, with 2.7 g/kg being more effective than 1.35 g/kg (42). In a subsequent study, it was confirmed that the positive effect is influenced by the size of AuNPs (spherical, 4 and 14 nm, i.t.), as significant RT enhancement was observed with 14 nm AuNPs, whereas 4 nm AuNPs did not exhibit RT enhancement (47). The study involving AuNPs modified with trastuzumab (spherical 30 nm, i.t.) confirmed the regression of tumor size (54), the same effect described was with nanoclusters with anti-RhoJ antibody (16.3 nm, i.v.) (74). Another efficient therapy for breast carcinoma was achieved by surface modification with the pH-responsive Au nanorods + doxorubicin (122 nm, i.t.), which demonstrated a synergistic effect in cancer chemo-radiotherapy (56) and nanoclusters with chitosan (53.1 ± 11.7 nm, i.v.) for improved tumor delivery (63). As well as combined radiotherapy and PTT using nanocages with HA (193.3 nm ± 8.1 nm, i.v.) and Nanocrystals with Se and Cu (17.6 ± 1.4 nm, i.v.) (90).

Studies on glioma models have shown that smaller particles (5.5 nm modified with FA, i.v.) were capable of detecting brain tissue (78). Studies with AuNPs modified with iRGD (penetrating peptide) + α-difluoro methylornithine (cell cycle regulator) (10.6 nm, i.v.) confirmed the effective blood–brain barrier penetration, target glioma cells with equal therapeutic effect compared to high-dose RT. The improved biodistribution and retention in the brain were due to surface modifications in this case (69). Also, a study with “bigger” 73 ± 10 nm AuNPs in the shape of nanodendalions (i.v.) confirmed the effect, but in nu/nu mice bearing subcutaneous C6 glioma tumor. Intraperitoneal application of AuNPs (spherical, 20 nm modified with insulin and chemotherapeutic cetuximab) for successful glioma therapy, is possible. Still, surface modification is necessary insulin for more targeted brain delivery and barrier penetration. This treatment is more effective than conventional chemoradiotherapy (temozolomide + RT) (75). Another success was demonstrated by the development of targeted sensitization-enhanced radiotherapy by diatomic SiAuNPs (187.1 ± 3.3 nm, intracranial application) modified on the surface of HA (targeted tumor delivery) and combined with 5-aminolevulinic acid (sonosensitizer), and the application of ultrasound prior to RT. This multimodal therapy caused an enhanced synergic therapeutic effect, allowed lower radiation doses, and improved survival in a glioblastoma multiforme model.

The exploration of gold nanostructures and their radiosensitizing potential has been extensive, driven by the need to surmount challenges and barriers for their successful integration into clinical trials. Based on the summarized characteristics of AuNPs and their possibilities, it can represent the step toward personalized medicine in radiotherapy.

## 5 Conclusion

Our comprehensive synthesis succinctly outlined the radiosensitizing potential of non-modified, modified, and combined AuNPs. The compiled data unequivocally highlights the substantial radiosensitizing impact across a spectrum of sizes and shapes of AuNPs when utilized with ionizing radiation or photothermal therapy (PTT). Notably, this effect can be further potentiated through surface modifications, offering many options for improved tumor targeting, accumulation, and enhanced therapeutic outcomes.

Main observations:

- Our study affirms the significant radiosensitizing effect of AuNPs when combined with radiotherapy.
- There is a noticeable gap in the literature regarding publications on large animal models, raising questions about the translatability of findings to clinical applications.
- Non-toxicity confirmation in many publications is limited to histological observations at the experiment’s end or a single measurement of hematological and biochemical indicators, often lacking differentiation of white blood cells.
- A comprehensive evaluation of non-toxicity should include more detailed tests, emphasizing the need for standardized assessments in the future studies.

These main observations underscore the promising potential of AuNPs in radiotherapy while emphasizing the importance of addressing gaps in research methodologies to enhance the reliability and applicability of findings in clinical settings. In conclusion, the current science landscape reflects a golden era in the realm of radiosensitization options within radiation therapy, where advancements in nanotechnology hold the key to transformative and personalized therapeutic strategies. In the end, we can conclude that there is an outgoing golden era in radiosensitization options in radiation therapy.

## Data Availability

The original contributions presented in the study are included in the article/supplementary material, further inquiries can be directed to the corresponding author.

## Glossary

8-HQ: 8-hydroxyquinoline
8-HQ-AuNPs: 8-hydroxyquinoline gold nanoparticles
AS1411: Guanine-rich DNA aptamer
AS1411-AuNPs: Guanine-rich DNA aptamer gold nanoparticles
Au: Gold
AuNPs: Gold nanoparticles
AuNPs-T: Gold nanoparticles coated with trastuzumab
AuNTs: Gold nanotriangles
BSA: Bovine serum albumin
cRAD: Arginine-alanine-aspartic acid
cRAD-AuNPs: Arginine-alanine-aspartic acid gold nanoparticles
cRGD: Arginine-glycine-aspartic acid
cRGD-AuNPs: Arginine-glycine-aspartic acid gold nanoparticles
Cs-AuNPs-ICG: Gold nanoparticles nanoclusters coated with fluorescent green dye and chitosan
CTX-INS-AuNPs: Insulin-coated and cetuximab antibodies gold nanoparticles
Cu: Copper
DA: Diazirines
DCNPs: Lanthanide-doped down conversion nanoparticles
DCNPs: Lanthanide-doped down conversion nanoparticles
DMMA: 2,3-dimethyl maleic anhydride
DOX: Doxorubicin
EPR: Enhanced permeability and retention
FA: Folic acid
FA-AuNPs: Folic acid gold nanoparticles
Fe: Iron
FeNPs: Iron nanoparticles
GTH: Glutathione
GTH-AuNPs: Glutathione gold nanoparticles
HA-AuNPs: Hyaluronic acid-coated gold nanocages
H-AuNPs: Histidine gold nanoparticles
HER2: Human epidermal growth factor receptor 2
HIF-1α: Hypoxia-inducible factor 1-alpha
IR: Ionizing radiation
iRGD: 9-amino acid
iRGD-AuNPs: 9-amino acid gold nanoparticles
LEV: Levonorgestrel
LEV-AuNPs: Levonorgestrel gold nanoparticles
Mn: Manganese
NBTXR3: Crystalline hafnium oxide nanoparticles
NIR: Near-infrared laser
NPs: Nanoparticles
Pd: Palladium
PdAuNPs: Palladium gold nanoparticles
PEG: Polyethylene glycol
NBPs: Nanobipyramids
PSMA-1: Prostate membrane specific antigen
PSMA-19-AuNPs: Prostate membrane specific antigen gold nanoparticles
PTT: Photothermal therapy
PVP: Polyvinyl pyrrolidone
RES: Reticuloendothelial system
RhoJ: Ras homolog family member J
Rhoj-AuNPs: Ras homolog family member J gold nanoparticles
ROS: Reactive oxygen species
RS: Radiosensitizer
RT: Radiotherapy
Se: Selenium
SeAuNPs: Selenium gold nanoparticles
SiNPs: Silica nanoparticles
siRNA: Small interfering RNA
siRNA-AuNPs: Small interfering RNA gold nanoparticles
SphK1: Sphingosine kinase 1 gene
SphK1-AuNPs: Sphingosine kinase 1 gene gold nanoparticles
SPIONs: Superparamagnetic iron oxide nanoparticles
TAT: Transactivating transcriptional activator
TAT-AuNPs: Transactivating transcriptional activator gold nanoparticles
Ti: Titanium
TiAuNPs: Titanium gold nanoparticles
TiNPs: Titanium nanoparticles
Tio: Tiopronin
Tio-AuNPs: Tiopronin gold nanoparticles
Zn: Zinc
ZnMnAuNps: Zinc-manganese gold nanoparticles
